# Roles of Therapeutic Bioactive Compounds in Hepatocellular Carcinoma

**DOI:** 10.1155/2021/9068850

**Published:** 2021-10-31

**Authors:** Divya Jain, Yogesh Murti, Wasi Ullah Khan, Rajib Hossain, Mohammad Nabil Hossain, Krishn Kumar Agrawal, Rana Azeem Ashraf, Muhammad Torequl Islam, Pracheta Janmeda, Yasaman Taheri, Mohammed M. Alshehri, Sevgi Durna Daştan, Balakyz Yeskaliyeva, Aliya Kipchakbayeva, Javad Sharifi-Rad, William C. Cho

**Affiliations:** ^1^Department of Bioscience and Biotechnology, Banasthali Vidyapith, Rajasthan, India; ^2^Institute of Pharmaceutical Research, GLA University, Mathura, India; ^3^Key Laboratory for Sustainable Utilization of Tropical Bioresource, College of Tropical Crops Hainan University, Haikou, China; ^4^Department of Pharmacy, Life Science Faculty, Bangabandhu Sheikh Mujibur Rahman Science and Technology University, Dhaka, Bangladesh; ^5^College of Chemistry, Chemical Engineering and Biotechnology, Donghua University, Shanghai, China; ^6^Faculty of Pharmacy, R.B.S. Engineering Technical Campus, Bichpuri, Agra, India; ^7^School of Pharmaceutical Science and Technology (SPST), Tianjin University, China; ^8^Phytochemistry Research Center, Shahid Beheshti University of Medical Sciences, Tehran, Iran; ^9^Pharmaceutical Care Department, Ministry of National Guard-Health Affairs, Riyadh, Saudi Arabia; ^10^Department of Biology, Faculty of Science, Sivas Cumhuriyet University, 58140 Sivas, Turkey; ^11^Beekeeping Development Application and Research Center, Sivas Cumhuriyet University, 58140 Sivas, Turkey; ^12^Faculty of Chemistry and Chemical Technology, Al-Farabi Kazakh National University, 050040 Almaty, Kazakhstan; ^13^Department of Clinical Oncology, Queen Elizabeth Hospital, Kowloon, Hong Kong, SAR, China

## Abstract

Hepatocellular carcinoma (HCC) is due to poor prognosis and lack of availability of effective treatment. Novel therapeutic strategies will be the fine tuning of intracellular ROS signaling to effectively deprive cells of ROS-induced tumor-promoting events. This review discusses the generation of ROS, the major signaling their modulation in therapeutics. We explore some of the major pathways involved in HCC, which include the VEGF, MAPK/ERK, mTOR, FGF, and Ser/Thr kinase pathways. In this review, we study cornerstone on natural bioactive compounds with their effect on hepatocarcinomas. Furthermore, we focus on oxidative stress and FDA-approved signaling pathway inhibitors, along with chemotherapy and radiotherapy enhancers which with early evidence of success. While more in vivo testing is required to confirm the findings presented here, our findings will aid future nonclinical, preclinical, and clinical studies with these compounds, as well as inspire medicinal chemistry scientists to conduct appropriate research on this promising natural compound and their derivatives.

## 1. Introduction

Globally, cancer is the major cause of mortality and morbidity, which can affect almost every organ in the human body [1]. According to the WHO, 1 out of 6 persons die due to cancer. In 2040, it will rise up to 29.4 million cancer cases globally per year [2]. Hepatocellular carcinoma (HCC) is one of the most lethal cancers; in men, it is the fifth, and in women, it is the eighth foremost cause of cancer death worldwide [3, 4]. There are several ways to inhibit liver cancer such as antioxidant [5], antiproliferative [6], anti-invasive [7], apoptotic [8], antimutagenic [9], anticarcinogenic [10], antitumor [11], and cytotoxic activity [12].

Nature is the big source of natural medicine and compounds derived from plants, animals, marines, and microbes [13–18]. Among them, plants provide many novel anticancer compounds [19] such as alkaloids [20, 21], flavonoids [22, 23], glycosides [24], saponins, tannins [25], and terpenoids [26] which are found from a plant having antioxidant and anticancer properties in a various cancer cell line, especially in a liver cancer cell line. HCC development is a multistep process that may include the alteration in host gene expression, DNA methylation, loss of heterozygosity, and point mutation, but still, we are lacking to determine the rate limiting step for initiation and progression of HCC [27].

As of late, improved information on oncogenic forms and the signaling pathways that manage tumor cell multiplication, differentiation, angiogenesis, invasion, and metastasis has prompted the recognizable proof of a few potential restorative focuses on that have driven the advancement of molecularly focused on treatments [28]. These medications which act straightforwardly on segments of the signaling pathways can control tumorigenesis and have demonstrated clinical advantage in patients with different tumor types. Here, we reviewed significant molecular signaling pathways embroiled in the pathogenesis of HCC and phytochemicals that are involved in the treatments as of now being developed and endorsed for HCC [29].

## 2. Signaling Pathways Involved in HCC

HCC carcinogenesis is a complex multistep process that involves a variety of signaling cascades at the molecular level. The major signaling pathways include vascular endothelial growth factor (VEGF) pathway, mitogen-activated protein kinase (MAPK/ERK) pathway, Wnt/*β*-catenin pathway, phosphatidylinositol-3 kinase (PI3K)/AKT/mammalian target of rapamycin (mTOR) signaling pathway, fibroblast growth factor (FGF) pathway, enzymes reactions generating ROS in liver cancers, enzymatic cycle of P450, mitochondrial dysfunction and signaling, and serine/threonine kinase (AKT) pathway.

### 2.1. VEGF Signaling Pathway

**Figure 1:**
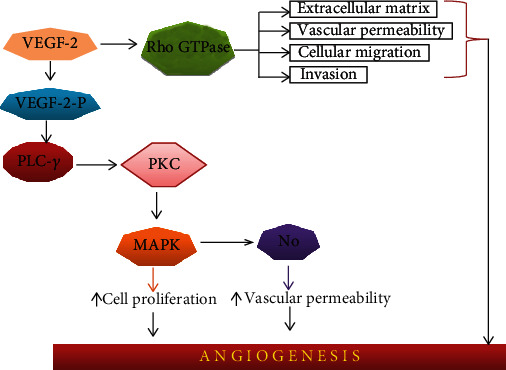
VEGF signalling mechanism. VEGF: vascular endothelial growth factor; PLC-*γ*: phospholipase C gamma; PKC: protein kinase C; MAPK: mitogen-activated protein kinase; NO: nitric oxide.

VEGF is a critical growth factor for angiogenesis during hypervascular HCC cancer development [30]. Deep located tumor cells are required to locate 100-200 *μ*m to acquire oxygen and nutrients for survival and proliferation. Tumor size greater than 2 mm^3^ required angiogenesis [31]. The major members of the family are VEGF-A, VEGF-B, VEGF-C, VEGF-D, and PIGF, and their potential forms include VEGF-A121 and VEGF-A165. VEGF has three main subtypes: VEGFR-1, VEGFR-2, and VEGFR-3. They all are embedded in the cell membrane; the extracellular region has a single TM seven immunoglobulins like domains and an intracellular region having a split tyrosine kinase domain [32]. They get phosphorylated after the ligand binding that activates PLC-*γ*, which leads to activate the PKC leading to the MAPK signaling pathway and activates endothelial NO that promotes cell proliferation and vascular permeability (Figure 1). It also activates the Rho GTPase [33, 34].

Out of all, VEGFR-2 seems to have a significant job in interceding practically the entirety of the known cell reactions to VEGFs [35]. The initiation of VEGFR-2 prompts endothelial cells to bring about their multiplication, relocation, expanded endurance, and advances vascular penetrability, whereas VEGFR-3 is significant for the lymphangiogenesis [36]. The articulation of VEGF mRNA in liver tumors was found in a larger part of HCC patients. The rule course of HCC dispersal and metastasis is through the entry vein in the liver, and VEGF mRNA level related well with portal vein tumor thrombus (PVTT) development of HCC. Immunohistochemical recognized high VEGF articulation is very much separated from HCC just as regions encompassing the HCC tissues [37]. The most immediate proof supporting the job of the VEGF pathway in HCC originated from late advancement in treatment hindering this pathway.

Bevacizumab (anti-VEGF monoclonal antibodies) are being tested for HCC [38], whereas sorafenib is capable of targeting vascular endothelial growth factor receptor 2 (VEGFR-2) and other proteins to inhibit the tumor angiogenesis [39]. In two significant clinical trials, it has been reported that in the late stage, sorafenib was effective in improving the outcomes of HCC patients.

### 2.2. Mitogen-Activated Protein Kinase Signaling Pathway

The mammalian mitogen-activated protein kinase (MAPK) family has three members, extracellular signal-regulated kinase (ERK), c-Jun NH_2_-terminal a kinase (JNK), and p38 that are involved in a variety of cellular activities [40]. Among them, the ERK pathway is involved in promoting cell proliferation, migration, survival, and tumor progression. In the ERK pathway ligand bind with receptor tyrosine kinase (RTKs), this triggers the tyrosine kinase domain activation [41]. That acts as docking sites for GRB2 and SOS proteins. This leads to an activation cascade of small GTPase RAS, Ser/Thr kinase RAF, and MEK [42].

**Figure 2:**
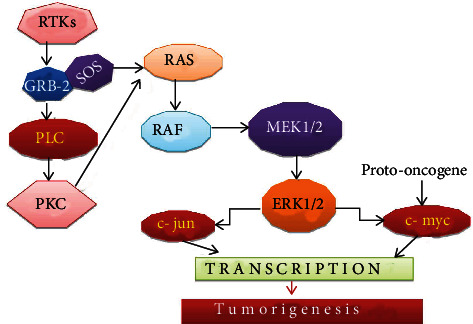
Mitogen-activated protein kinase signaling mechanism. RTK: receptor tyrosine kinase; GRB-2: growth factor receptor bound protein-2; SOS: Son of Sevenless; RAF: rapidly accelerated fibrosarcoma; PLC: phospholipase C; PKC: protein kinase C.

ERK activation can alter the various activities of transcription factors and gene expression level which leads to alteration in cell cycle progression [43]. The phosphorylated ERK (ERK-P) can activate c-myc that regulates cell growth and cell proliferation (Figure 2) [44, 45]. Moreover, it also promotes the survival of cancer cells by regulating the BIM and MCL1 apoptotic pathways [46, 47]. The role of the ERK pathway in HCC is confirmed by AZD6244 (MEK inhibitors) that block cell proliferation and promote programmed cell death in liver carcinoma [48, 49].

Another MAPK signaling pathway includes JNK (JNK-1 and JNK-2) activated by MKK-4 and MKK-7, and downstream substrates include c-Jun [50]. There is strong evidence that JNK-1 increased histone H3 lysine 4 and 9 trimethylations, tumor size that results in the upregulation of cell growth promoting genes [51]. Unlike ERK/JNK pathways, p38 are induced by MKK-3, 4, and 6 and have a suppressive role in HCC [52]. The mechanism behind the p38 activity is the suppression of JNK and the negative regulation of cell proliferation [53].

### 2.3. Wnt/*β*-Catenin Signalling Pathway

**Figure 3:**
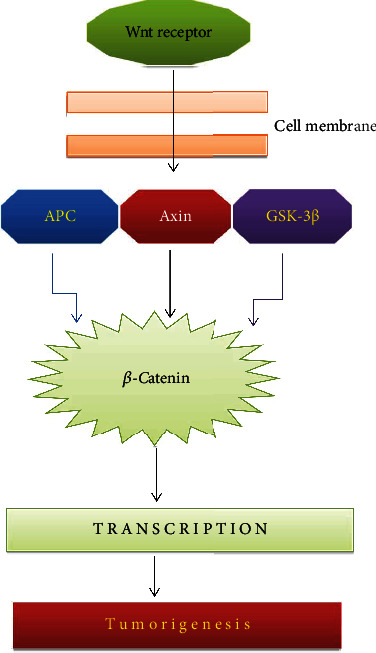
Wnt/*β*-catenin signaling pathway. APC: adenomatous polyposis coli; GSK3*β*: glycogen synthase kinase 3 beta.

Wnt ligands are cell surface ligands that play a significant role in normal liver function. They form complexes with Frizzled receptors and LRP-5/LRP-6 coreceptors. *β*-Catenin forms a complex with various tumor suppressor proteins like APC, axin, and Ser/Thr kinase GSK3*β* in which APC and axin proteins make structural changes in GSK3*β* to phosphorylate *β*-catenin [54]. This leads to *β*-catenin destruction in the cytosol. Upon Wnt binding with a ligand, axin is enlisted to the film to LPR-5, and the *β*-catenin demolition complex is then inactivated [55]. This permits the unphosphorylated *β*-catenin to aggregate and to move into the nucleus (Figure 3). This *β*-catenin then structures a complex with a TCF-LEF group of DNA restricting record variables to initiate the TCF-LEF target gene [56]. A significant number of the objective gene is engaged with cell multiplication, i.e., cyclin D1.

Phosphorylated *β*-catenin binds with E-cadherin and performs cell to cell adhesion that is a significant process in the development of tumor metastasis. The researcher found strong evidence of Wnt/*β*-catenin's role in liver carcinoma. In most of the HCC cases, the level of *β*-catenin is overexpressed that leads to accumulation and results in cell proliferation and inhibiting differentiation. There are likewise considered partner *β*-catenin transformations or initiation with compounded HCC result, i.e., bigger tumor size, expanded vascular intrusion, and point mutation or deletion [57].

Such predominant addition of work transformations normally happens at the N-terminal phosphorylation destinations on *β*-catenin, including the locales phosphorylated by GSK3*β* that control *β*-catenin debasement. Changes at these positions disturb acknowledgment by GSK3*β* bringing about increasingly stable *β*-catenin protein. In this manner, different reasons for *β*-catenin aggregation may exist [58].

It has been demonstrated that the pharmacologic restraint of *β*-catenin diminishes the endurance of hepatoma cells. Inactivation of *β*-catenin silencer APC prompted the unconstrained improvement of HCC in a mice model, recommending the immediate commitment from actuated Wnt motioning to hepatocarcinogenesis. In any case, parts of the Wnt pathway may speak to potential restorative intercession that focuses on rewarding HCC [59].

### 2.4. Phosphatidylinositol-3 Kinase (PI3K) Signaling Pathway

A phosphatidylinositol-3 kinase (PI3K)/AKT/mammalian target of rapamycin (mTOR) signaling pathway has a class to the large group of related kinases that have two subunits, i.e., catalytic and regulatory. It is an intracellular signal transducer enzyme that can phosphorylate the -OH group of phosphatidyl-inositol. The p85 is a regulatory subunit of PI3K that can interrelate with phosphor-tyrosines on activated RTKs that recruit the ligands to the plasma membrane and initiates the enzymatic activities. The lipid second messenger phosphatidyl-inositol-triphosphate (PIT) is activated by PI3K in response to activating the PI3K. The downstream ligand of PI3K is AKT kinase having domain on C terminus that is pleckstrin homology (PH), which binds with PIT and phosphorinositide-dependent kinase 1 (PKDK1) [60].

PDK1 activates the AKT kinase activity that phosphorylates various proteins and manages cellular activities. The downstream effector of AKT is mTOR that belongs to the PI3K family that contains FAT-FATC domains, FRB, and catalytic kinase domains [61]. The AKT phosphorylates the TSC1/TSC2 that activates the Rheb, a small G-protein that finally activates the mTOR for its cellular activity protein translation. Excessive protein translation generally results in abnormal cell growth and tumorigenesis. The negative regulator of this signaling pathway is PTEN that dephosphorylates the PIT [62].

In HCC pathogenesis, reduced PTEN expression has been linked with high recurrence rate, tumor stage, and low survival rate. In the treatment policy, the PI3K/AKT/mTOR signaling pathway is upregulated, and inhibitors could play an important role. In addition, everolimus [Afinitor, RAD-001 (40-O-(2-hydroxyethyl)- rapamycin)] is a rapamycin analog (rapalog) mTOR inhibitor administered per oral and has been approved by FDA that showed a significant reduction in tumor growth rate by downregulating gene expression which related to ribosomal protein S6 kinase beta-1 (S6K1) and eIF4E-binding protein (4EBP) suppression and inhibits signaling downstream [63].

### 2.5. Fibroblast Growth Factor Pathway

**Figure 4:**
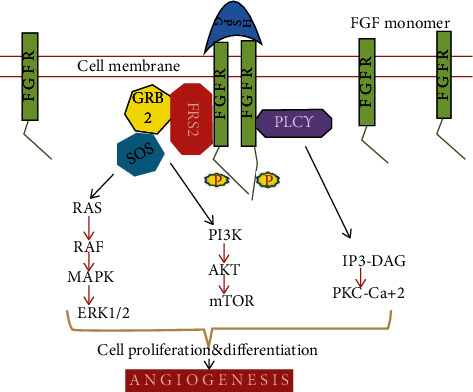
Fibroblast growth factor signaling pathway. GBR: growth factor receptor-bound; SOS: Son of Sevenless; PLC*γ*: phospholipase C gamma; MAPK: mitogen-activated protein kinase; ERK1/2: extracellular signal-regulated kinase; PI3K: phospho-inositide-3-kinase; AKT: protein kinase B; mTOR: mammalian target of rapamycin; PKC-Ca^2+^: protein kinase C-Ca^2+^.

Fibroblast growth factor (FGF) ligand is a family of 20 different ligands that consist of an extracellular transmembrane domain and intracellular tyrosine kinase domain that is associated with tumorigenesis [64]. Many studies suggested the significant role of FGF in the progression of chronic hepatitis. The tyrosine kinase domains after dimerization can activate the different intracellular signaling pathways [65]. FGF-substrate-2 (FRS-2) is an important adaptor of the FGF receptor that can recruit various proteins like SOS and GRB2 after the phosphorylation to activate RAS-GTPase which promotes the different downstream signaling like Wnt, MAPK, and PI3K/AKT pathways as shown in Figure 4 [66]. The downstream signaling of FGF leads to carcinogenesis via angiogenesis. The overexpression of FGFR1 significantly accelerates the growth of HCC in the mouse model [67]. FGF8, 17, and 18 increase the HCC cell survival, and suggesting a role in the progression of HCC, likewise, FGF15 also promotes hepatocellular proliferation in mice that also contribute towards the HCC development. In addition to this, epithelial-to-mesenchymal transition is promoted by FGFR19 that functions via the GSK3*β* signaling pathway through FGFR4 stimulation [68].

### 2.6. Enzyme (P450) Reactions Generating ROS in Liver Cancers

Human cytochrome P450s are one of the major sources of ROS. It plays a very important role in maintaining cellular redox species balance which is mandatory for cell signaling and normal cellular functions like an immune response [69]. Normal redox balance is very important for normal organ functioning so that any malfunction cannot lead to various ailments like oxidative stress, aging, and carcinogenesis. Likewise, ROS and RNS can also disrupt biological functions, which lead to cellular damage and oxidative stress [70]. In most cases, variations in structure patterns of lipids, nucleic acids, and proteins are the main targets of ROS. Oxygen radicals by some nonenzymatic oxidation of arachidonic acid form F_2_-isoprostanes through lipid peroxidation [71]. These F_2_-isoprostanes not only show their biological effects but are also used as alternate markers to measure ROS levels and oxidative stress [72]. Human CYP2E1 has known to produce ROS through the process of lipid peroxidation, and their products interact with DNA and cause DNA adducts [73], whereas protein modifications through ROS are also possible particularly amino acid cysteine modification can cause downstream signaling in toxic pathways leading to carcinogenesis especially HCC.

**Figure 5:**
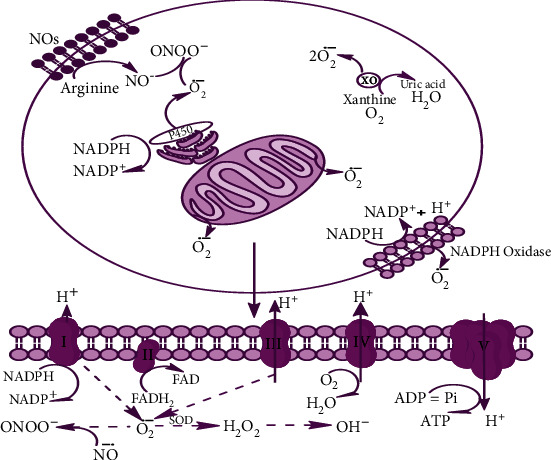
Generation of reactive oxygen species (ROS).

ROS are generated in the mitochondria, peroxisomes, cytochrome p450, and other components of the cell [74, 75]. Initially, an electron is provided to O_2_^−^ that further dismutases to H_2_O_2_. Here, superoxide dismutase converts O_2_ to H_2_O_2_ which is a stable molecule and can cross membranes. In the cytochrome chain, free radicals are formed when the electrons, donated by FADH and NADH, react with oxygen and other electron acceptors [76] as shown in Figure 5.

### 2.7. Enzymatic Cycle of P450

**Figure 6:**
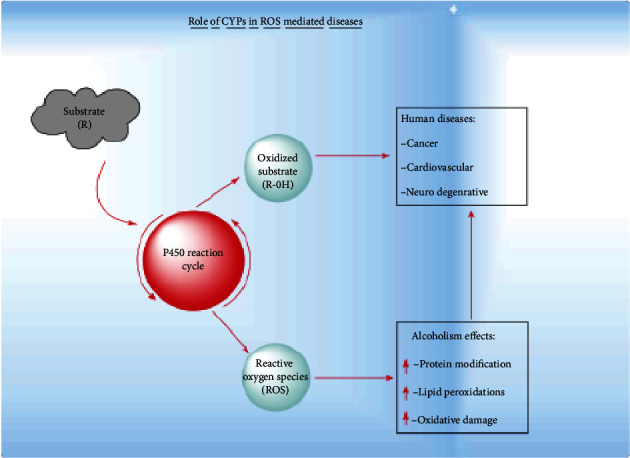
Cytochrome P450 contribution to human diseases caused by ROS and produced as a result of substrate metabolism by CYP 450s which cause elevations in protein and nucleic acid levels and cause lipid modifications. These modified products further lead to lipid peroxidation processes and also cause DNA damage which in turn causes cancer.

**Figure 7:**
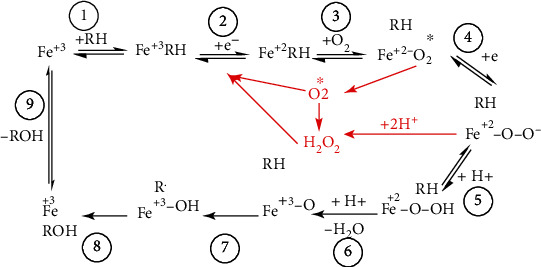
Summarized CYP 450 catalytic cycle. Catalytic cycle of P450s showing some critical steps where ROS are generated (shown in red). These ROS can further cause damage to cellular components that lead to various diseases.

Human cytochrome P450s (CYP) are a superfamily of monooxygenases that are primarily known for the oxidation of the vast majority of xenobiotics in phase I metabolism helping in increasing substrate polarity and helping in excretion [77]. CYP generates ROS and how they contribute to an increase in oxidative stress. In the very first step, the substrate (R-H) binds to the active site of the CYP enzyme via ferric iron (Fe^3+^) of the heme thiolate group (Figure 6). In the second step, the heme thiolate group receives one electron from the NADPH regenerating system and CPR cytochrome peroxidase reductase (redox partner of CYP enzyme) and gets reduced to Fe^+2^. This is the time when molecular oxygen binds to O_2_ and CPR, then donates the second electron and reduces the Fe^2+-^O_2_ complex which activates oxygen in the complex (Fe^2+-^O_2_^−^). In the next step (H^+^), ions get into the active site by some special ion channels and cleave the O-O bond and release water. The complex (FeO_2_^+3^) then removes a proton in the step from the substrate and leaves an intermediate RFe^3+^.OH^−^. In the last step, the –OH hydroxyl group is transferred to the substrate radical and the oxidized substrate is released at the end (Figure 7). The latest research is underway to specifically highlight the role of intermediate species in various types of CYP-mediated oxidation reactions; these intermediate species are formed during steps of the CYP 450 catalytic cycle [78]. Oxygen concentration and pH are the two major factors that play an important role in CYP-mediated coupling reactions [79].

In this way, the CYP-mediated ROS-generated reaction through their catalytic cycle modifies the cellular components which lead to various diseases. It is clear how CYP during their catalytic cycle alters the redox reactions and disrupts the normal P450 catalytic cycle, which results in oxidative stress leading to development of various kinds of disease.

### 2.8. Mitochondrial Dysfunction and Signaling

**Figure 8:**
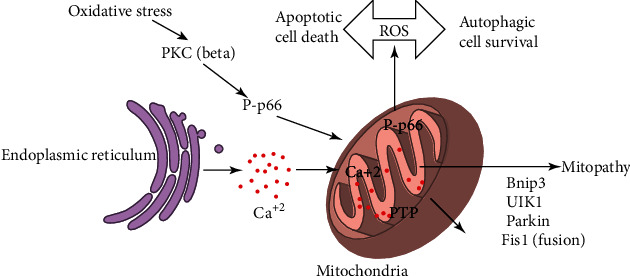
Signaling pathways regulating mitochondrial function.

Mitochondria regulate the urea cycle, amino acid, iron, and fat metabolism and produce energy required for the cell to perform all important functions [80–82]. In cells, the major site for the production of ROS is mitochondrion [83]. Increased levels of ROS production act as a clear death threat to the cells because it directly affects the defense mechanism, the most exclusive autophagy, and plays their role as signaling molecules which ultimately results in cell death either by autophagy or apoptotic pathway (Figure 8). In each case, mobilization of various H_2_O_2_ sensitive pathways is initiated [84]. Moreover, in starved conditions, autophagy process increases due to elevated ROS production by mitochondria [85].

Similar studies in obese (ob/ob) mice have also shown increased production of FFAs from glucose, elevated mt ROS productions, and elevated levels of triglycerides [86], higher oxidative stress due to increased lipid peroxidation while decreased hepatic mitochondrial components of MRC and decreased ATP levels [87]. All these mitochondrial changes require alterations in mitochondrial ROS levels, changes in mitophagy, biogenesis, and relevant signaling pathways of ROS.

It also requires changes in cholesterol and GSH levels in mitochondria. Changes in FFAs, lipid peroxidation products, and TNF are observed as well [88]. Oxidative stress causes ROS generation which results in the activation of cascades involving PKC*β*-dependent phosphorylation of pp66shc and its movement to the matrix of mitochondria, and these mitochondria are also the main target of ROS.

### 2.9. Serine/Threonine Kinase (AKT) Pathway

It is also known as protein kinase B (PKB) play an important role in angiogenesis in pathological condition and tumor growth via different Ser/Thr kinase family members like liver kinase B1 (LKB1), calcium/calmodulin-dependent protein kinase IV (CAMKIV), and sulfatase (SULF2). LKB1 has multiple phenotypic expressions for the regulation of cell polarity, metabolism, proliferation, and apoptosis. In HCC, LKB1 phosphorylate at Ser428 that phosphorylates AMP-activated protein kinase (AMPK) microtubule affinity-regulating kinase (MARK) phosphorylation leads to activation and localization of cofactors like pseudokinase Ste20-related adaptor (STRAD*α*) and the scaffolding protein MO25. The STRAD*α* and MO25 form a complex that binds with the LKB1 and relocalize it from nucleus to cytoplasm and stimulate its cell proliferation and angiogenesis [89]. Calcium (Ca^+2^) regulates various biological processes as a second messenger via a variety of signaling pathways. It binds to downstream effector calmodulin (CAM) and increases the affinity toward calmodulin-kinase like Ca^+2±^/CAM-protein kinase-IV (CAMKIV). CAMKIV expression is increased in HCC and shows cell proliferation and cell cycle regulation [90].

**Figure 9:**
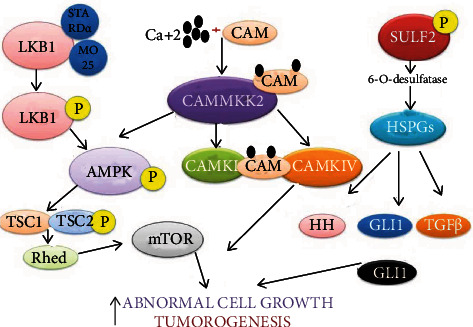
Serine/threonine kinase (AKT) pathway. LKB1: liver kinase B1; HSPGs: heparin sulfate proteoglycans; CAMKK2: Ca+2/CAM-dependent protein kinase kinase-2; CAMKIV: calcium/calmodulin-dependent protein kinase IV; SULF2: sulfatase 2; STRAD*α*: Ste20-related adaptor; HH: hedgehog; GLI1: GLI family zinc finger 1.

Overexpression of calcium in HCC binds with the calmodulin that forms a complex with upregulated Ca^+2^/CAM-dependent protein kinase kinase-2 (CAMKK2). Further, this complex stimulates the CAMKIV and AMPK that leads to stimulate angiogenesis [91]. Sulfatase 2 (SULF2) is elevated in HCC that is linked with increased tumor growth, hepatoblast phenotype, and a higher rate of tumor recurrence. Dephosphorylation of SULF2 enzyme leads to 6-O-desulfurase that acts on heparin sulfate proteoglycans (HSPGs) and releases the cytokines and growth factors like inflammatory mediators. These mediators regulate the SULf2-directed tumorigenesis via different pathways like hedgehog (HH), WNT, and TGF*β*. These pathways transcripts the common pathway GLI family zinc finger 1(GLI1). SULF2-GLI1 promotes tumor growth via heterodimerization of STAT3 that work via the JAK/STAT signaling pathway as shown in Figure 9 [92]. Many signaling pathway inhibitors have been approved by the FDA or are in clinical trials (as shown in Table 1).

## 3. Antioxidant Effect of Medicinal Plants

Reactive oxygen species (ROS) is a class of reactive molecules, which are generated from oxygen metabolism [105]. Furthermore, several damages occurred in cells and tissues, not only during infections but also various degenerative disorders including cardiovascular disease, aging, neurodegenerative diseases, and cancer by ROS [106, 107]. For radical detoxification, human cells have defense mechanisms. In these cells, superoxide dismutase (SOD) transforms superoxide into hydrogen peroxide and oxygen, then converted the H_2_O_2_ into water, and toxic ROS are scavenged by catalase (CAT), glutathione peroxidase (GPx), and reduce oxygen-free radicals in cells. Additionally, antioxidant enzymes (SOD, CAT, and GPx), along with vitamin A, C, E play a provital role in the antioxidant defense mechanisms [108–111]. In recent years, researchers focused on the natural phytochemicals found in berry crops, teas, herbs, oilseeds, beans, fruits, and vegetables, which are the potential sources of antioxidant compounds to treat several [19, 112–119].

## 4. Oxidative Stress Associated with HCC

Oxidative stress happens once there is an associate degree imbalance between reactive chemical element species (ROS) generation and attenuated by antioxidant enzymes or compounds. Excessive production of ROS will cause aerophilous harm to biomacromolecules leading to supermolecule peroxidation and carcinogenesis [120, 121].

Anticancer medication increases malondialdehyde (MDA) level and decreases inhibitor enzymes like GPx, GR, CAT, SOD, and GSH [122, 123]. They increase (MDA) and XO level, cytokines TNF-*α*, IL-6, i-NOS, cyclooxygenase-2, and P38-MAPK, NF-*κ*B, and generation of ROS and RNS (reactive nitrogen species) in a viscous cell [124, 125]. In ethanol, the cytoplasm and mitochondria are reborn aldehyde and acetate by vasoconstrictive, ALDH, NAD+, and NADH, which may increase ROS generation in the liver cell, cause DNA harm, mitochondrial pathology, lipid peroxidation, supermolecule denaturation, and stimulate many viscous sicknesses as well as steatosis, fibrosis, cirrhosis, steatohepatitis, and carcinoma [126].

Ordinarily, ROS and RNS are generated by strongly bound enzymes. Too much stimulation of NAD(P) H and negatron transport chain results in the production of ROS, which leads to stress and can injure the cell structures, lipids, proteins, and DNA. The production of ROS by vegetative cells was originally referred to as “the metabolism burst” because of the redoubled consumption of chemical elements by these cells. This method is catalyzed by NAD(P)H enzyme and is important for the disinfectant action of phagocytes [127]. The metabolism of organic compounds (L-arginine) forms NO∙ free radicals. The gas synthase (NOS) enzymes are catalyzing the process, and through 5 electron oxidization of a guanidine gas of L-arginine, it converts L-arginine into L-citrulline and NO∙ radical [128].

## 5. Potential of Phytochemicals

### 5.1. Scavenging of ROS

In any kind of cancer, lethal effects due to oxidative stress can be harmful. To counterbalance this, antioxidant mechanisms in normal human cells should be needed [129]. Besides, it can be considered as a significant process that is taken by phytoconstituents to prevent cancer (Table 2).

The scavenging process is done by different antioxidant mechanisms so that they could not be able to cause any disfigurement. Some nonradical and radical ions that work as ROS are hydrogen peroxide (H_2_O_2_), superoxide radical (O_2_^−^), hydroxyl radical (.OH), and peroxyl radical (ROO.). Protein, DNA, and lipids are excessively harmed due to these ions and also altered the regular cellular functioning system. Some phytochemicals act as antioxidant or prooxidant whose modulators are mainly guided by two factors, one is the microenvironment and another is the concentration of ROS present within the cells. To keep a healthy balanced metabolic activity, the proper quantity of ROS in normal cells is necessary. Now, cells can be damaged through oxidative stress, especially when ROS comes from extrinsic sources. Normal cells can be injured by external factors. In this case, phytoconstituents can play an important role as antioxidants to protect those cells from damage [171].

### 5.2. Phytochemicals Evaluation in Clinical Trials

Phytochemicals derived from medicinal herbs that are now clinically tested and used in the treatment of liver fibrosis are Inchin-ko-to (TJ-135), Yi Guan Jian, Fufang-Liu-Yue-Qing, and DangguiBuxue Tang [172–175]. An enormous number of anticancer compounds, which are now in progress, nevertheless lead to clinical studies in their initial phases. Through various preclinical researches, the efficacy of different phytochemicals has represented such as, andrographolide, berberine, capsaicin, curcumin, genistein, ursolic acid, and withaferin A.

Andrographolide restrains tumor development by obstructing tumor adjustment to hypoxic conditions. The detected effects of andrographolide (100 mg/kg) were attributed to the restriction of the hypoxia-inducible figure (HIF) [176]. The recovery rate of different myeloma patients has made strides through the use of andrographolide in a clinical trial [177, 178]. While investigations are undertaken via the large quantity of information on preclinical efficacy, clinical studies are limited in the evaluation of berberines and andrographolide genuine potential as a carcinoma operator [179].

Capsaicin supplementation significantly reduced the establishment of preneoplastic foci in a rat model of hepatocarcinogenesis caused by diethylnitrosamine, HCC cell lines were likewise stopped from proliferating, and apoptosis was triggered on a dose-based manner [180, 181]. Furthermore, HCC cells were shown to be more sensitive than normal hepatocytes to capsaicin induced cytotoxicity suggesting that it might have a chemotherapeutic effect [182].

Curcumin is a chemopreventive agent with a lot of optimism. This has prompted clinical practices to investigate the pharmacokinetics and effectiveness of curcumin in patients. It was shown to be safe and nontoxic in phase I clinical studies, even at large dosages (8 g/day). However, it had limited absorption individuals [183, 184]. Their clinical trials, either alone or as anticancer agent combinations, demonstrated efficacy, despite challenges to bioavailability, in several disease sites [185–187].

The medication with genistein (140 mg/kg) is by preventing aberrant nuclear *β*-catenin harvests and concealing WNT signaling features [188]. Ursolic acid (UA) was represented to upgrade the restorative impacts of oxaliplatin in the mouse model of CRC by restraining the tumor and expanding the survival rate. Tumor shape is lessened by the UA nanoparticles by focusing on caspases and p53 with downregulation of Bcl-2 and cIAP, instigating apoptosis and driving to cervical cancer cell distortion [189], whereas the tumor development of human colorectal carcinoma (HCT-116) cells which overexpress AKT and microvessel arrangement is hindered through the verbal organization of Withaferin A (5 mg/kg) in a mouse model [190].

### 5.3. Detoxification of Enzymes

Xenobiotic compounds are responsible for putting impacts on humans affecting tissues. To lessen that impact, there are several responses or potentials [191]. Among them, the initialization of some detoxifying enzymes is important, especially for the liver [192]. Detoxified enzymes can be induced by antioxidative phytochemicals found in plants. These phytochemicals mainly target antioxidant response or electrophile response elements (ARE/EpRE) to modulate the molecular pathways. These pathways mainly depend on three main components like ARE, nuclear factor erythroid 2 p45-related factors 2 (Nrf2), and Kelchlike ECH-associated protein 1 (Keap1) [193].

### 5.4. Modification of Genomic Stability

Phytochemicals targets both DNA repair and their damage mechanism, where genomic stability within cells plays an important role. Besides, this genomic stability also helps a lot when chemopreventive agents trigger a selective number of cancer cells [194, 195]. There are some therapeutic agents which generate the DNA repair pathways in normal cells to modulate the stress conditions within the cells. In a contradicting way, DNA damage response can also be increased when cancer cells are exposed in a large number. As a result, apoptosis can happen, and cells can be dead permanently [196].

### 5.5. Cancer Cell Metabolism

Cell metabolism in tumors plays an important role in the stimulation process in protooncogenes by involving ROS production [197, 198]. The survival and growth rate of tumor cells largely depend on their metabolic requirements which are adjusted by themselves [199]. Energy requirements are supplied by glucose, and thus, it initiates tumor growth. Besides glucose, glutamine also plays an important role in tumor growth by providing nitrogen for the biosynthesis process. Phytochemicals can obstruct basal transport of glucose [200], e.g., curcumin can convert glucose to glutathione [201].

### 5.6. Chemotherapy and Radiotherapy Enhancers

Cancer and complications that are associated with it are treated by chemotherapy and radiotherapy for decades. Radiotherapy began in the twentieth century before chemotherapy, as the primary treatment of cancer. Approximately 8% of total cancer patients need radiotherapy, but unfortunately, it causes acute toxicity even at low doses of radiation. To overcome these side effects and prevention of resistance to chemotherapy, a promising new approach is developed by the scientist by using the medicinal plant-derived drugs like taxol to combat against cancer; many researches is going on radioprotectors and radiosensitizers [202]. Radioprotectors are the compounds that are used to protect the normal cell during radiotherapy sessions; on the other hand, radiosensitizers are the molecule that is used to sensitize the tumor cell and increases the efficiency of cancer therapy. Some plants like *Pilea microphylla* act as a radioprotector and prevent the depleting SOD, GSH, CAT, and thiol levels during the radiotherapy in HCC.  Table 3 shows the effect of medicinal plants and their bioactive compounds in chemotherapy and radiotherapy [203–206].

## 6. Conclusion and Future Perspective

The scavenging property of plant-derived bioactive compounds should be the pathway to treat HCC as they block the propagation stage in oxidative chain reactions. Furthermore, these compounds inhibit cancer cell growth and act as potent anticancer agents via different cellular and molecular mechanisms. At present, a plethora of kinase inhibitors against specific molecular targets are being investigated in HCC, which initiate differential networks that consequently result in HCC cell cycle promotion. It offers hope that other effective therapies will eventually be developed.

## Figures and Tables

**Table 1:** Clinical trials and FDA approved molecules that exert inhibitory effect for each signaling pathway.

Compounds/drugs	Chemical structure	Clinical trial/FDA approved	Receptor/target	Description	Inhibitor	References
Vatalanib (PTK787/ZK 222584)	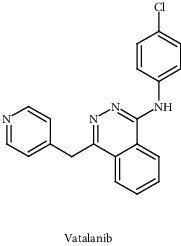	Phase -III	VEGFR1, VEGFR2, VEGFR3, PDGFR-*β*, c-Kit	Small-molecule tyrosine kinase receptor inhibitor	VEGF signaling pathway	[93]
AE-941 (Neovastat®)	Structure not available	Phase -III	VEGF–VEGFR-binding MMP2, MMP9	Shark-cartilage component	VEGF signaling pathway	[93, 94]
Sorafenib	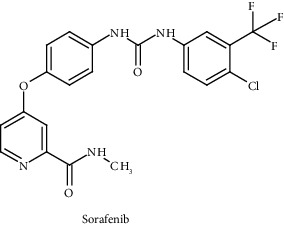	Phase -III	VEGFR-2, PDGFR-*β*, FLT3, c-Kit	Small-molecule Raf kinase and tyrosine kinase inhibitor	VEGF signaling pathway	[93]
Trametinib	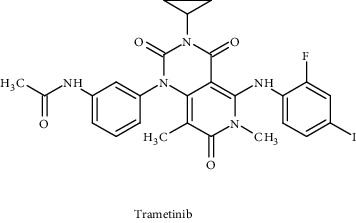	FDA approved	BRAF	Allosteric, non-ATP competitive small-molecule inhibitors	MAPK pathway	[95, 96]
Binimetinib	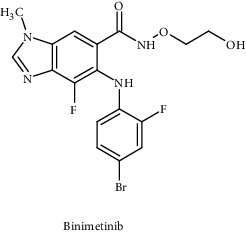	FDA approved	BRAF^V600E^ or BRAF^V600K^	Allosteric, non-ATP competitive small-molecule inhibitors	MAPK pathway	[96]
Genistein	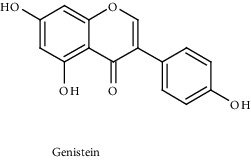	Phase I-II	GSK3-*β*	Inactivate Wnt signaling by upregulating the expression of GSK3-*β* and E-cadherin	Wnt/*β*-catenin signalling pathway	[97]
PRI-724	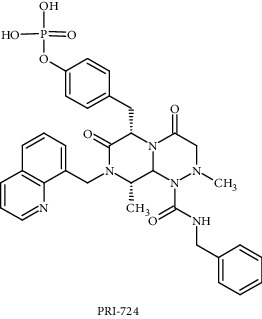	Phase 1	*β*-Catenin	Blocks the interaction between *β*-catenin and its transcriptional coactivator CREB-binding protein (CBP)	Wnt/*β*-catenin signalling pathway	[98, 99]
Idelalisib	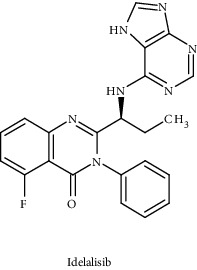	USFDA approved	PI3K-*δ*	Capable of inducing apoptosis and inhibit AKT phosphorylation and downstream effectors	PI3K signaling pathway	[100, 101]
Duvelisib	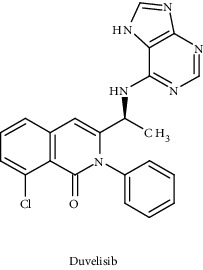	USFDA approved	PI3K-*γ* and PI3K-*δ*	Capable of inducing apoptosis and inhibit AKT phosphorylation and downstream effectors	PI3K signaling pathway	[101, 102]
Erdafitinib	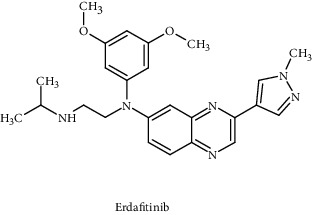	USFDA approved	FGFR1-4	Inhibits tumor cell differentiation, proliferation, angiogenesis	Fibroblast growth factor pathway	[103]
Netarsudil	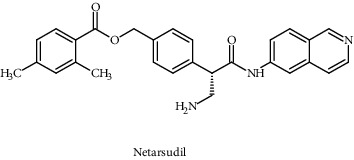	USFDA approved	ROCK1/2 nonreceptor	Inhibits the enzyme rho kinase	Serine/threonine kinase (AKT) pathway	[104]

**Table 2:** Bioactive compounds and their anticancer effect on hepatocarcinoma.

S. no.	Phytomolecules	Animal/cell lines	Methods	Chemical structure	Mechanisms	Ref.
1.	Andrographolide (labdane diterpene)	Swiss albino mice	*In vivo* (diethylnitrosamine) antioxidant assay	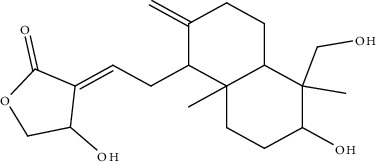	1. Liver biochemical parameters 2. Increased MDA and NO level 3. Decreased level of Gal-3 and IL-6	[130]
2.	Allicin (organosulfur compound)	HCC xenograft tumors in nude mice	*In vivo*	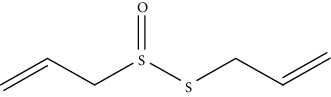	1. Increased intracellular ROS level 2. Reduced MMP 3. Activated caspase-3 and PARP 4. Downregulated Bcl-2	[131]
3.	Aloe emodin (antraquinone)	HepaRG cells	MTT assay, annexin V-FITC/PI	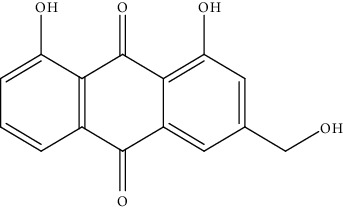	1. Significant reduction in cell viability after treatment 2. Induction of apoptosis in HepaRG cell line 3. Provoked ROS generation 4. Depolarization of MMP 5. Induced cell cycle in S-phase 6. Increases the level of mitochondrial cytochrome C, Fas, p21, Bax/Bcl2, and p53	[132]
4.	Arbutin (glycosylated hydroquinone)	Mice	*In vivo* (X-ray irradiation)	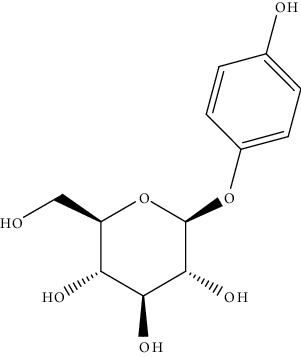	Liver biochemical parameters like ALP, ALT, and AST were significantly reduced	[133]
5.	Berberine (benzyl-isoquinoline alkaloid)	Hep3B, BEL-7404	Cell counting kit-8 assay and EdU assay	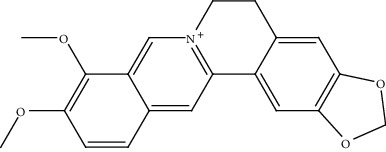	It suppressed the glutamine uptake by inhibiting SLC1A5	[134]
6.	Boldine (alkaloid)	Wistar rat	*In vivo* (diethylnitrosamine)	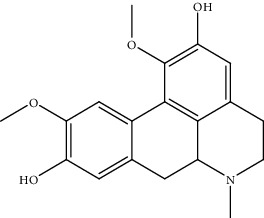	1. It induced the apoptosis 2. Upregulate the protein expression of Bax and cleaved caspase 3	[135]
7.	Betulinic acid (triterpene)	NOD/SCID mice, HepG2, LM3, MHCC97H	MTT assay, pulmonary metastasis model	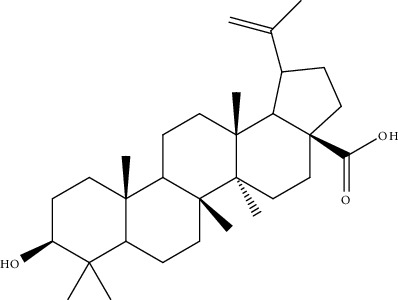	1. Induction of apoptosis 2. Increased the Bax and cleaved caspase-3 3. Decreased the level of Bcl-2 4. Decreased the level of ROS 5. Inhibit metastasis *via* MMP-2, MMP-9, and TIMP2	[136]
8.	Capsaicin (homovanillic acid alkaloid)	SCLC (NCI-H69, NCI-H82, DMS53, DMS114), chicken eggs	MTT assay, BrdU and PCNA proliferation assays, CAM assay, nude mice models, Chromatin immunoprecipitation (ChIP) assay	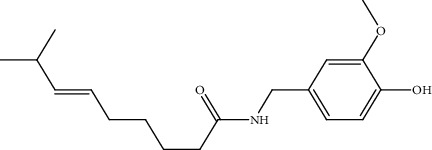	1. Decreased expression of E2F-responsive proliferative genes (cyclin E, thymidylate synthase, cdc25A, and cdc6, both at mRNA and protein levels) 2. G1 phase arrest	[137]
9.	Caffeine (purine alkaloid)	HepG2, HLF, Huh7, PLC/PRF/5	Hoechst 33258 staining, MAPK activity, flow cytometry	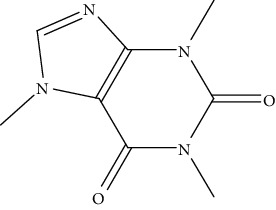	1. Inhibited the cell proliferation 2. Activated the MERK-regulating kinase and ERK pathway 3. Downregulation of EGRF	[138]
10.	Crocin (apocarotenoid)	Male albino, Wistar rats, HepG2	*In vivo* (DEN) induced hepato-carcinogenesis	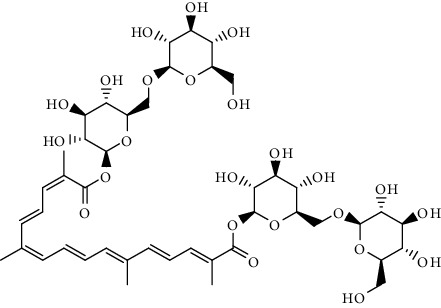	1. Antiproliferative 2. Induced proapoptotic body 3. Arresting the cell cycle at S and G2/M phases 4. Induced apoptosis	[139]
11.	Coumarin-6-sulfonamides (sulfonamide derivative)	HepG2	Sulforhodamine B (SRB) method, annexin V–FITC apoptosis assay	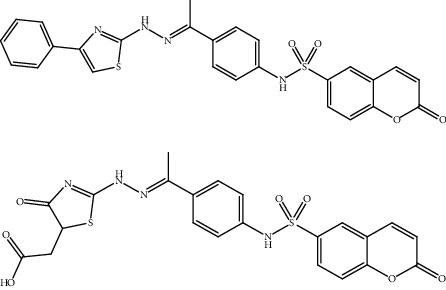	1. Induced apoptosis 2. Upregulation of the Bax 3. Downregulation of the Bcl-2 4. Increased caspase-3 levels 5. Arrest in the G2-M phase	[140]
12.	Carnosic acid (polyphenolicditerpene)	B16F10 cell xenograft model	MTT assay, BrdU incorporation assay	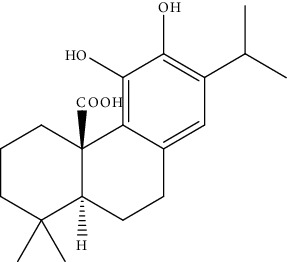	1. Arrested G0/G1 phase 2. Enhances p21 expression 3. Reduces the values of AST and ALT	[141]
13.	Curcumin (diarylheptanoid)	Liver cancer stem cells (LCCs), HepG2	MTT assay, Western blot analysis	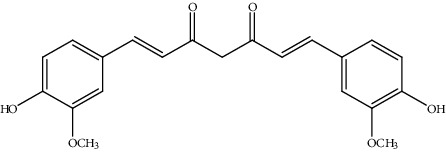	1. Inhibited cell proliferation 2. Induced apoptosis 3. Inhibited the activation of the PI3K/AKT/mTOR signaling pathway	[142]
14.	Daidzein (7-hydroxyisoflavones)	SK-HEP-1	TUNEL assay	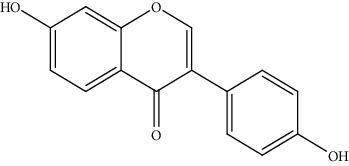	1. Increased expression of prdx-3 2. Decreased ROS level 3. Upregulation of Bak protein 4. Downregulation of Bcl-2 and BclxL proteins 5. Increased the release of mitochondrial cytochrome c 6. Activated the APAF-1, caspase 9, and caspase 3	[143]
15.	Embelin (benzoquinone)	Male Swiss mice, Wistar albino rats, Sprague Dawley rats	*In vivo* (N-nitrosodiethylamine, CCl_4_)	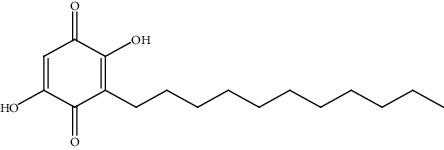	1. Decreased incidence of preneoplastic foci 2. Decreased biochemical markers (SGOT, SGPT, ALP, GGT, GST, and LPO)	[144]
16.	Esculetin (coumarin derivative)	C57BL/6J mice Hepa1-6 cells	*In vivo* MTT assay	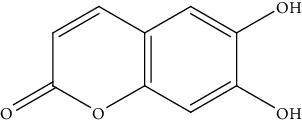	1. Inhibited proliferation of HCC cells 2. Arrest cell cycle at S phase 3. Induced apoptosis 4.Increased caspase-3 and caspase-9 activity 5. Increased Bax expression 6. Decreased Bcl-2 expression	[145]
17.	Emodin (anthraquinone)	HepG2 Hep3B Huh7 SK-HEP-1 PLC/PRF5	Western blotting, quantitative real-time PCR, tumor xenograft assay, Ki67 cell proliferation assay, annexin-V staining, luciferase assay	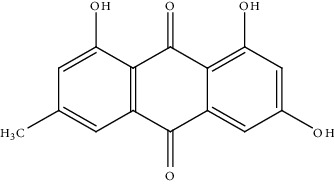	1. Attenuated cholesterol synthesis and oncogenic AKT signaling 2. Inactivated STAT3 3. Cell cycle arrest in the G1 phase	[146]
18.	Emodinsuccinylester (trihydroxyanthraquinone derivative)	BALB/c nu/nu athymic nude mice, Hep3B, Huh7	Western blot, quantitative RT-PCR, xenograft mouse model	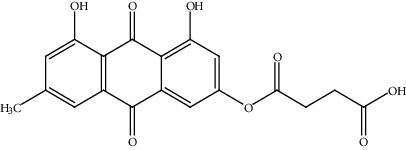	1. Inhibited HCC cell proliferation and migration 2. Decreased transcription level and protein expression of androgen receptor 3. Enhanced of zeste homolog 2	[147]
19.	(-)-Epigallocatechin-3-gallate (catechin)	Mice, Hep3B, He-pG2, SK-hep1, HCC-LM3, Huh7, SMMC7721	Western blot analysis, cell viability analysis, tumor xenograft in nude mice model	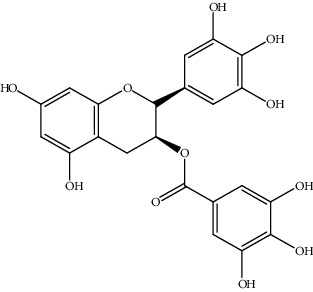	1. Inhibited Hep3B cells by both antiproliferation and proapoptosis 2. ER*α*36-EGFR-Her-2 feedback loop, PI3K/Akt, and MAPK/ERK pathways were inhibited	[148]
20.	Genistein (isoflavone)	Hepa1-6 cell line	Cell viability assay, Flow cytometry	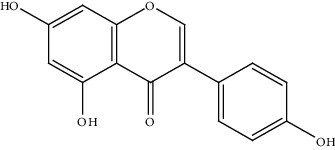	1. Inhibited the growth of Hepa1-6 cells 2. Induced apoptosis	[149]
21.	Gallic acid (phenolic acid)	Wistar albino rats	*In vivo* (diethylnitrosamine)	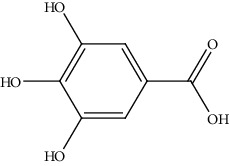	1. Decreased the size of tumors 2. Decreased the levels of marker enzymes in serum 3. Decreased the levels of AgNORs and PCNA	[150]
22.	18𝛽-Glycyrrhetinic acid (triterpene)	HepG2, H22	MTT assay	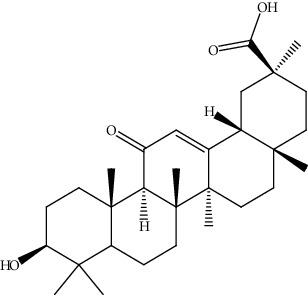	Decreased cell viability in higher concentration	[151]
23.	Hesperidin (flavonoid)	HepG2 cells	MTT assay, DAPI staining	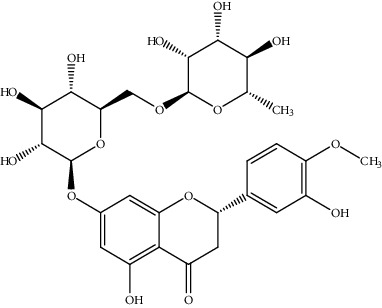	1. Induced cell death 2. Activated mitogen-activated 3. Protein kinase ERK1/2 4. Inhibited cell proliferation 5. Arrested G1 phase cells 6. Induced proptosis like cell death 7. Induced depletion of MMP	[152]
24.	Honokiol (biphenol neolignans)	CCA cell lines (KKU-100 and KKU-213)	MTT assay	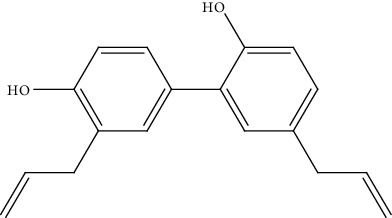	1. Inhibited cell proliferation 2. Arrested G0/G1 cell cycle phase 3. Induced apoptosis 4. Suppressed the adhesion and migration of cell 5. Inhibited the MMP-9 and MMP-2 activity	[153]
25.	Kaempferol (Flavonoid)	HepG2 cell	Cell counting kit-8 assay, BrdU incorporation assay, Guava Nexin assay, two-chamber migration (invasion) assay	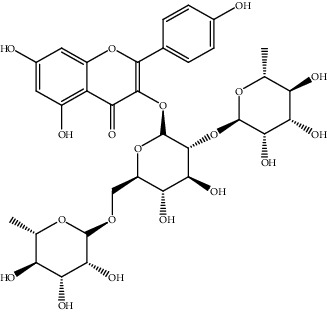	1. Inhibited cell proliferation, migration, and invasion 2. Induced cell apoptosis 3. Reduce the expression of miR-21 4. Enhanced the expression of PTEN 5. Inactivated PI3K/AKT/mTOR signaling pathway	[154]
26.	Lupeol (triterpene)	MHCC-LM3, nude mice (BALB/c-nu/nu)	MTT assay, xenograft model	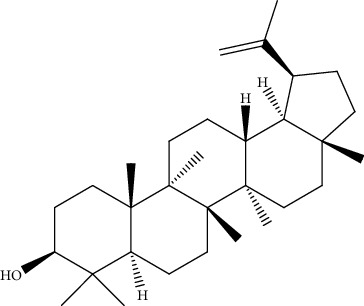	1. Inhibited *in vivo* tumorigenicity 2. Downregulated CD133 expression 3. Sensitize (PTEN)-Akt-ABCG2 pathway	[155]
27.	Magnolol (lignan)	HepG2 cells, nude mice (Balb/c nu/nu)	MTT assay, Transwell assays, flow cytometric analysis	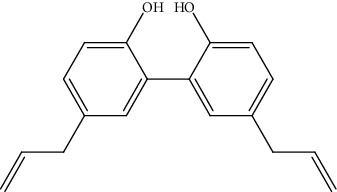	1. Inhibited the proliferation, migration, and invasion of cells 2. Induced apoptosis 3. Induction of ER stress 4. Release of cytochrome C 5. Arrested S-phase 6. Suppressed tumor growth *in vivo*	[156]
28.	Mangiferin (xanthone glucoside)	Male Swiss albino mice	*In vivo* (lead-induced) MTT assay	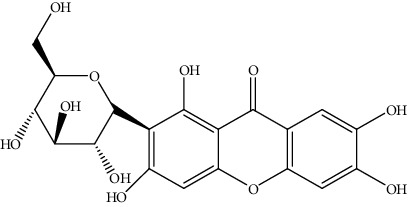	1. Decreased ROS formation 2. Restored the MMP 3. Regulation of Bcl-2/Bax 4. Inhibited activation of MAPKs (phospho-ERK 1/2, phosphor-JNK phospho- p38) 5. Induced apoptosis	[157]
29.	Naringenin (trihydroxyflavanone)	HepG2 cells	Flow cytometry Cell viability assay	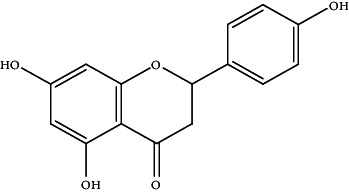	1. Inhibited the cell proliferation 2. G0/G1 and G2/M phase arrest 3. Induced apoptosis 4. Increased ratio of Bax/Bcl-2 5. Release of cytochrome C 6. Activation of Caspase 3	[158]
30.	Oleuropein (monoterpenoid)	HepG2, Huh7	Cell counting kit 8, flow cytometric analysis, cell viability assay, luciferase assay	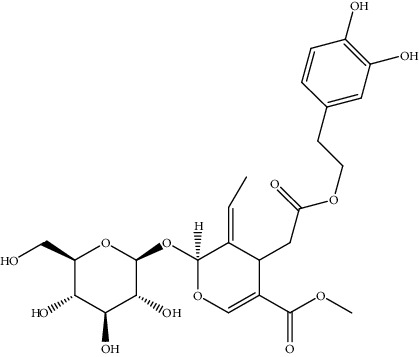	1. Suppressed expression of activated AKT 2. Inhibited cell growth 3. Induced cell apoptosis 4. Inhibited PI3K/AKT signaling pathway	[159]
31.	Oleanolic acid (triterpenoid)	HepG2	MTT assay, cell viability assay, annexin V-FITC	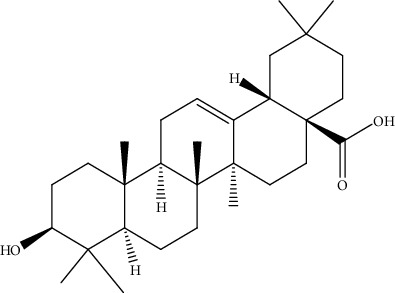	1. Induced cytotoxic effect 2. G0/G1 cell cycle arrest 3. Reduced MMP	[160]
32.	Parthenolide (sesquiterpene lactone)	HepG2	MTT assay, DAPI, TUNEL staining, Western blotting, monodansylcadaverine (MDC), AO staining	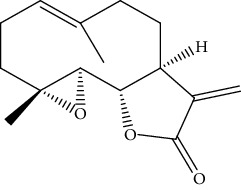	1. Increased the number of apoptotic nuclei 2. Reduced expression of Bcl-2 3. Increased expression of Bax, p53, and caspase-3 and 9 4. Induced autophagy 5. Inhibited the expression of the Ki-67 gene	[161]
33.	Phycocyanin (phycobiliproteins)	NSCLC (A549, NCI-H1299, NCI-H460, and LTEP-A2)	MTT assay, annexin V-FITC and 7AAD staining	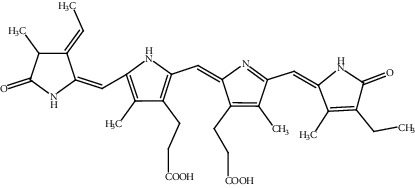	1. Induced apoptosis 2. Regulated the NF-kB signaling of NSCLC cells 3. Significantly reduced the expression of MMP-2 and MMP-9 4. Suppressed the proliferation of NSCLC cells 5. Arrested G1 and S phase of cell cycle	[162]
34.	Quercetin (flavonoid)	HCC (LM3), nude mice model	Flow cytometry, TUNEL assay, qRT-PCR, Western blotting	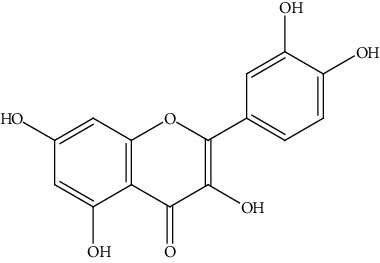	1. Suppressed cell viability 2. Induced cell apoptosis 3. Inhibited the activation of the JAK2/STAT3 pathway 4. Inhibited cell migration and invasion 5. Inhibited tumor growth in nude mice model	[163]
35.	Rutin (flavonoid)	HepG2 cell line	Diamino-benzoic acid and bromodeoxyuridine assays, lactate dehydrogenase leakage assay, fluorimetric assay, dichlorofluorescein assay, northern blot	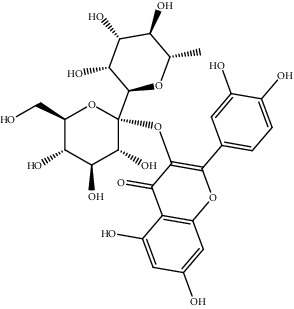	1. Decreased ROS and MDA concentration 2. Arrested cell growth at higher concentration 3. No cytotoxic effect on HepG2 cells	[164]
36.	Rosmarinic acid (coumaric acid derivative)	H22 tumor-bearing mice	ELISA, Western blotting, qRT-PCR	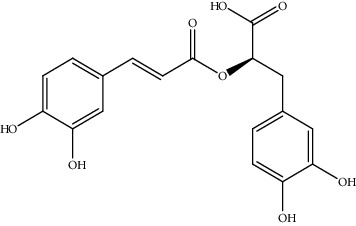	1. Decreased p65 phosphorylation 2. Inhibited the tumor growth 3. Decreased the elevated level of cytokines	[165]
37.	Resveratrol (polyphenol)	HepG2	MTT assay, flow cytometric analysis, Western blot analysis, laser confocal microscopy	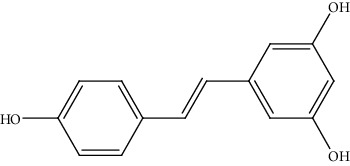	1. Inhibited cell proliferation 2. Arrested G1 phase 3. Downregulated the expression of cyclin D1, p38 MAP kinase, Akt, and Pak1 4. Increased ERK activity 5. Induced apoptosis	[166]
38.	Salidroside (*p*-hydroxy-phen-ethyl-*β*-d-glucoside)	Human hepatocellular carcinoma (HHCC)	MTT assay, Western immunoblotting	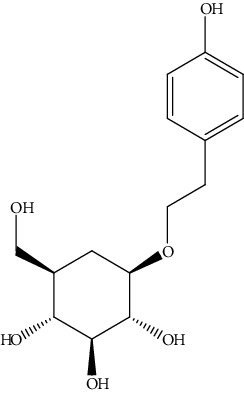	1. Inhibited cell proliferation 2. Arrested G2 phase 3. Inhibited CDK-cyclin activity	[167]
39.	Ursolic acid (pentacyclictriterpenoid)	HepG2, Hep3B	Cytotoxicity assay, ethidium homodimer assay, deoxynucleotidyl transferase-mediated dUTP nick-end labeling assay, cell cycle analysis, Western blotting	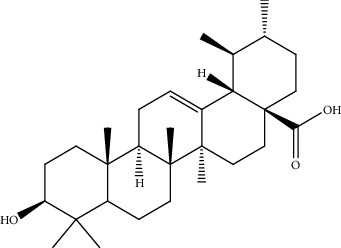	1. Enhanced the expression of PARP and Caspase3 2. Increased the sub-G1 population 3. Attenuated the expression of AEG1gene 4. Increased the phosphorylation of AMPK, GSK3*β*, and coenzyme A 5. Attenuated the phosphorylation of AKT and mTOR	[168]
40.	Withaferin A (steroidal lactone)	Nude mice model, MHCC97, JHH-5	Xenogen *in vivo* imaging system, Western blot analysis, liquefied Matrigel assay	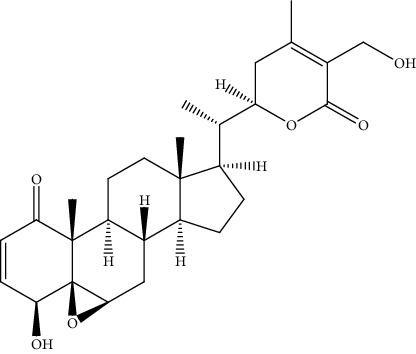	1. Inhibited the tumor growth 2. Decreased intrahepatic metastasis 3. Inhibited the expression of Pyk2, ROCK1 protein, and VEGF 4. Suppressed the formation of actin projection	[169]
41.	Xanthatin (sesquiterpene lactone)	HepG2, Bel-7402, SMMC-7721	MTT assay, cell viability assay, flow cytometry, annexin-V/PI double staining assay, Western blotting, immunofluorescence staining, dual-luciferase reporter gene assay	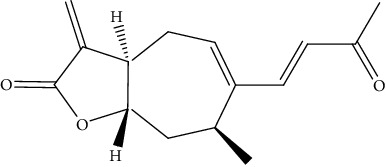	1. Reduced cell viability 2. Arrested S phase cell cycle 3. Induced apoptosis 4. Induced ERS and activated UPR pathway	[170]

**Table 3:** Medicinal plants and their bioactive compounds used in radiotherapy and chemotherapy.

*Plants as radioprotector/radiosensitizer in HCC*	
*Amaranthus paniculatus* Linn.	Decreases the depleted level of endogenic antioxidant enzymes during radiotherapy of mice liver.
*Coronopus didymus* (L.)	Enhance the level of antioxidant enzymes in the liver of mice.
*Grewia asiatica* L.	Augmented the SOD, CAT, and GSH levels in the liver of irradiated mice.
*Glycyrrhiza glabra* L.	It protected plasmid DNA and reduced the liver microsomal LPO level in rat from irradiation.
*Hypericum perforatum* L.	*In vitro* and *in vivo* studies show the increased level of SOD, CAT, GSH-Px, and GSH during radiation therapy
*Pilea microphylla* (L.) Liebm.	Increased level of endogenous antioxidant enzyme levels in the liver of mice.
*Rosmarinus officinalis* L.	Augmented the SOD, CAT, and GSH levels in blood and liver of mice during the radiation therapy.
*Xylopia aethiopica* (Dunal) A.Rich.	Protect the liver of rat from *γ*-radiation
*Bioactive phytomolecules as adjunct with chemotherapy*	
Curcumin	Used as an adjuvant with vinorelbine chemotherapy and enhances the antiproliferative effect of drugs.
Quercetin	Used as an adjunct in doxorubicin, busulfan, and cisplatin chemotherapy. It also increased cytotoxic effects of these drugs and protect from drug-induced nephrotoxicity.
Ginsenosides	Used as an adjunct with cisplatin and 5-FU chemotherapy and enhanced antiproliferative effect.

## Data Availability

The data used to support the findings of this study are available from the corresponding author upon request.
